# Development and Validation of a Prognostic Nomogram for Extremity Soft Tissue Leiomyosarcoma

**DOI:** 10.3389/fonc.2019.00346

**Published:** 2019-05-01

**Authors:** MingFeng Xue, Gang Chen, JiaPing Dai, JunYu Hu

**Affiliations:** Department of Orthopaedics, The Second Hospital of Jiaxing, The Second Affiliated Hospital of Jiaxing University, Jiaxing, China

**Keywords:** extremities, leiomyosarcoma, nomograms, predictor, prognosis

## Abstract

**Background:** Extremity soft tissue leiomyosarcoma (LMS) is a rare disease with a poor prognosis. The aim of this study is to develop nomograms to predict the overall survival (OS) and cancer-specific survival (CSS) of patients with extremity soft tissue LMS.

**Methods:** Based on the Surveillance, Epidemiology, and End Results (SEER) database, 1,528 cases of extremity soft tissue LMS diagnosed between 1983 and 2015 were included. Cox proportional hazards regression modeling was used to analyze prognosis and obtain independent predictors. The independent predictors were integrated to develop nomograms predicting 5- and 10-year OS and CSS. Nomogram performance was evaluated by a concordance index (C-index) and calibration plots using R software version 3.5.0.

**Results:** Multivariate analysis revealed that age ≥60 years, high tumor grade, distant metastasis, tumor size ≥5 cm, and lack of surgery were significantly associated with decreased OS and CSS. These five predictors were used to construct nomograms for predicting 5- and 10-year OS and CSS. Internal and external calibration plots for the probability of 5- and 10-year OS and CSS showed excellent agreement between nomogram prediction and observed outcomes. The C-index values for internal validation of OS and CSS prediction were 0.776 (95% CI 0.752–0.801) and 0.835 (95% CI 0.810–0.860), respectively, whereas those for external validation were 0.748 (95% CI 0.721–0.775) and 0.814 (95% CI 0.785–0.843), respectively.

**Conclusions:** The proposed nomogram is a reliable and robust tool for accurate prognostic prediction in patients with extremity soft tissue LMS.

## Introduction

Soft tissue leiomyosarcoma (LMS) is an aggressive sarcoma, which originates from smooth muscle cells (1). Soft tissue LMS accounts for about 5–10% of all soft tissue sarcomas (2). It occurs in different sites, including the retroperitoneum, intraabdominal sites, and extremities (3). Extremity LMS comprised about 10–15% of extremity sarcomas, with a preference for the lower limb (4–6). Extremity LMS tends to have a better prognosis than uterine, retroperitoneal, or major vessel LMS (1, 7), but worse overall survival (OS) than other soft tissue sarcoma subtypes (8). Moreover, patients with metastasis usually have a poorer prognosis (2, 9, 10). Current treatment for extremity LMS includes systemic chemotherapy along with surgery and/or radiation therapy for local control. Surgical resection is the most widely accepted treatment modality (11). Significant independent predictors for survival in LMS patients are primary site, age, tumor location, tumor size, margin status, and histological grade (2, 12–14), whereas abdominal site, tumor size >15 cm, positive resection margin and higher histological grade predict poor survival. Further, tumor size and margin status were independent predictors of local recurrence, while tumor size and grade were independent predictors for metastasis in LMS patients (13). However, there have been few studies exploring the prognostic factors of extremity LMS.

The nomogram is recognized as a practical clinical tool to predict survival outcome for many tumors by incorporating numerous predictors (15–17), helping clinicians and patients to estimate the probability of OS and cancer-specific survival (CSS) and make decisions. The excellent predictive accuracy, strong robustness, and user-friendliness of nomograms have made them a new standard to guide the management of cancer patients (18). However, no prognostic nomogram has been established for extremity LMS. Therefore, this study aimed to develop a user-friendly tool that could be used for reliable estimation of survival and to guide individualized management decisions.

## Materials and Methods

### Study Population

A case-listing session procedure was used to obtain patient data from the Surveillance, Epidemiology, and End Results (SEER) Program (19). Patient data were obtained from SEER^*^Stat 8.3.5 Database: Incidence—SEER 18 Regs Custom Data, Nov 2017 Sub (1973–2015 varying)—Linked to County Attributes—Total U.S., 1969–2016 Counties, National Cancer Institute, Division of Cancer Control and Population Sciences (DCCPS), Surveillance Research Program, released April 2018, based on the November 2017 submission. Using the International Classification of Diseases for Oncology, 3rd edition (ICD-O-3), we identified all patients with extremity soft tissue LMS (ICD-O-3 histologic type: 8890, Leiomyosarcoma, not otherwise specified [NOS]; 8891, Epithelioid leiomyosarcoma; 8896, Myxoid leiomyosarcoma; ICD-O-3 site code: C49.1, upper limb; C49.2, lower limb). The SEER database is publicly available and contains no personal identification information.

The inclusion criteria were as follows: (1) confirmation of histologic type of LMS; (2) site limited to soft tissue extremity only, excluding bone sites; (3) age at diagnosis ≥18 years; (4) diagnosis between 1983 and 2015; (5) diagnosis acquired from histology; and (6) complete follow-up without missing data.

The exclusion criteria were as follows: (1) patients diagnosed based on clinical presentation, radiography, or autopsy; (2) patients with unknown grade, stage, tumor size, surgery, radiation treatment, survival time or follow-up status; (3) survival time <1 month.

Data extracted from the SEER database included age, gender, year of diagnosis, location, tumor grade, tumor stage, tumor size, surgical treatment, radiation treatment, cause of death, and survival time. Patient age of 60 was chosen as a cutoff because it was an indicator of older patients and a negative factor of survival (1, 13). Surgery or radiation treatment for tumors in our study refers to treatment for local primary tumors. OS was calculated as the time from diagnosis to death due to any cause, and CSS was calculated as the time from diagnosis to death attributable to this cancer (20).

### Statistical Analyses

The SPSS statistical software (version 21.0) was used to perform univariate and multivariate analysis via Cox proportional-hazard regression models, to determine the independent predictors of OS and CSS. Hazard ratios (HRs) and corresponding 95% confidence intervals (CIs) were used to reveal the effects of various factors on OS or CSS. Independent predictors (*p* < 0.05) from the training cohort were identified to construct the nomograms.

R version 3.5.0 (https://www.r-project.org/) was used to develop and validate the nomograms, using the rms and survival packages (17, 21). Discrimination and calibration curves were constructed both internally (training cohort) and externally (validation cohort) to predict the accuracy of the nomograms (22). Bootstraps with 1,000 resamples were applied to validate the nomograms internally and externally (22). The concordance index (C-index) was used to evaluate the discrimination between observed and predicted outcomes (23).

## Results

### Patient Characteristics

Demographic and clinicopathological characteristics of patients are shown in Table 1. A total of 1,528 patients diagnosed with extremity soft tissue LMS were identified from 1983 to 2015. Records of 1,528 patients were collected and randomly divided into training (*n* = 764) and validation (*n* = 764) cohorts. The mean and median age at diagnosis of all patients were 62 and 63 years, respectively. More than half (*n* = 815, 53.3%) of the patients were male. Most of the patients (*n* = 1,445, 94.6%) had surgical treatment. Of these 1,528 patients, 676 (44.2%) died and the median survival was 105.0 ± 7.0 months. The median age of the training cohort was 64 years and the median survival was 101.0 ± 10.3 months. The 5-year OS and CSS rates of the entire cohort were 61.7 and 70.0%, respectively. For the validation cohort, the median age was 63 years and the median survival was 108.0 ± 11.3 months. The 5-year OS and CSS rates were 62.4 and 71.2%, respectively.

**Table 1:** Baseline demographics and clinical characteristics of the patients with soft tissue extremity leiomyosarcoma.

**Category**	**All patients (*n* =1,528)**	**Training cohort (*n* = 764)**	**Validation cohort (*n* = 764)**
Mean age (years)	62	63	62
Median age (years)	63	64	63
**Age (Years)**			
<60	639 (41.8%)	307 (40.2%)	332 (43.5%)
≥60	889 (58.2%)	457 (59.8%)	432 (56.5%)
**Gender**			
Female	713 (46.7%)	339 (44.4%)	374 (49.0%)
Male	815 (53.3%)	425 (55.6%)	390 (51.0%)
**Year of Diagnosis**			
<2,000	210 (13.7%)	99 (13.0%)	111 (14.5%)
≥2,000	1,318 (86.3%)	665 (87.0%)	653 (85.5%)
**Location**			
Upper limb	387 (25.3%)	195 (25.5%)	192 (25.1%)
Lower limb	1,141 (74.7%)	569 (74.5%)	572 (74.9%)
**Tumor size**			
<5 cm	727 (47.6%)	355 (46.5%)	372 (48.7%)
5–10 cm	514 (33.6%)	251 (32.9%)	263 (34.4%)
>10 cm	287 (18.8%)	158 (20.7%)	129 (16.9%)
**Tumor Grade^a^**			
Low	642 (42.0%)	316 (41.4%)	326 (42.7%)
High	886 (58.0%)	448 (58.6%)	438 (57.3%)
**Distant Metastasis**			
No	1,390 (91.0%)	682 (89.3%)	708 (92.7%)
Yes	138 (9.0%)	82 (10.7%)	56 (7.3%)
**Surgical Treatment**			
Yes	1,445 (94.6%)	718 (94.0%)	727 (95.2%)
No	83 (5.4%)	46 (6.0%)	37 (4.8%)
**Radiation Treatment**			
Yes	730 (47.8%)	364 (47.6%)	366 (47.9%)
No	798 (52.2%)	400 (52.4%)	398 (52.1%)
**Dead**			
Yes	676 (44.2%)	353 (46.2%)	323 (42.3%)
No	852 (55.8%)	411 (53.8%)	441 (57.7%)
5-year OS rate ± SE	62.0 ± 1.4%	61.7 ± 1.9%	62.4 ± 2.0%
5-year CSS rate ± SE	70.6 ± 1.5%	70.0 ± 2.1%	71.2 ± 2.1%
10-year OS rate ± SE	46.5 ± 1.6%	45.5 ± 2.2%	47.5 ± 2.2%
10-year CSS rate ± SE	61.1 ± 1.7%	60.6 ± 2.5%	61.7 ± 2.5%
Median survival (months) ± SE	105.0 ± 7.0	101.0 ± 10.3	108.0 ± 11.3

a *Low: Grade I (well differentiated) and Grade II (moderately differentiated); High: Grade III (poorly differentiated) and Grade IV (undifferentiated anaplastic). OS, overall survival; CSS, cancer-specific survival; SE, standard error*.

### Independent Predictors for Patients With Extremity Soft Tissue LMS

In the training cohort, the univariate analysis showed that age, tumor grade, distant metastasis, tumor location, tumor size, and surgery were significantly associated with OS and CSS (*p* < 0.05) (Table 2). Additionally, the radiation treatment was significantly associated with CSS (*p* < 0.05) (Table 2). Multivariate analyses were employed to identify independent predictors of survival for patients with extremity soft tissue LMS (Table 3). Using multivariate analysis of all patients, age ≥60 years, high tumor grade, distant metastasis, tumor size ≥5cm, and surgery were found to be independent risk factors of both decreased OS and CSS.

**Table 2:** Univariate analysis of OS and CSS in the training cohort.

**Category**	**OS (Log-rank *p*-value)**	**CSS (Log-rank *p*-value)**
Age at diagnosis(<60 vs. ≥60)	< 0.001	< 0.001
Gender (female vs. male)	0.811	0.812
Year of diagnosis (<2,000 vs. ≥2,000)	0.134	0.435
Location (upper limb vs. lower limb)	0.007	0.001
Tumor grade^a^ (low vs. high)	< 0.001	< 0.001
Distant metastasis (yes vs. no)	< 0.001	< 0.001
Tumor size	< 0.001	< 0.001
>10 cm vs. <5 cm	< 0.001	< 0.001
>10cm vs. 5–10 cm	< 0.001	< 0.001
5–10 cm vs. <5 cm	< 0.001	< 0.001
Surgical treatment (yes vs. no)	< 0.001	< 0.001
Radiation treatment (yes vs. no)	0.154	0.024

a *Low: Grade I (well differentiated) and Grade II (moderately differentiated); High: Grade III (poorly differentiated) and Grade IV (undifferentiated anaplastic). OS, overall survival; CSS, cancer-specific survival*.

**Table 3:** Multivariate analysis for OS and CSS in the training cohort.

**Variable**	**OS**		**CSS**	
	**Hazard ratio** **(95% CI)**	***P*-value**	**Hazard ratio** **(95% CI)**	***P*-value**
**Age (Years)**				
<60	1		1	
≥60	2.770 (2.158–3.554)	<0.001	1.796 (1.318–2.447)	<0.001
**Location**				
limb	1		1	
Lower limb	0.938 (0.714–1.233)	0.647	1.051 (0.700–1.577)	0.811
**Tumor Grade^a^**				
Low	1		1	
High	1.956 (1.506–2.541)	<0.001	2.632 (1.695–4.088)	<0.001
**Distant Metastasis**				
No	1		1	
Yes	2.251 (1.649–3.072)	<0.001	3.759 (2.465–5.733)	<0.001
**Tumor Size**				
<5 cm	1		1	
5–10 cm	1.499 (1.143–1.966)	0.003	2.602 (1.696–3.991)	<0.001
>10 cm	2.705 (2.004–3.651)	<0.001	4.401 (2.756–7.028)	<0.001
**Surgical Treatment**				
Yes	1		1	
No	2.871 (1.922–4.291)	<0.001	2.218 (1.325–3.714)	0.002
**Radiation Treatment**				
Yes	NA		1	
No	NA	NA	0.973 (0.707–1.341)	0.870

a *Low: Grade I (well differentiated) and Grade II (moderately differentiated); High: Grade III (poorly differentiated) and Grade IV (undifferentiated anaplastic). OS, overall survival; CSS, cancer-specific survival*.

### Nomogram Construction and Validation

**Figure 1:**
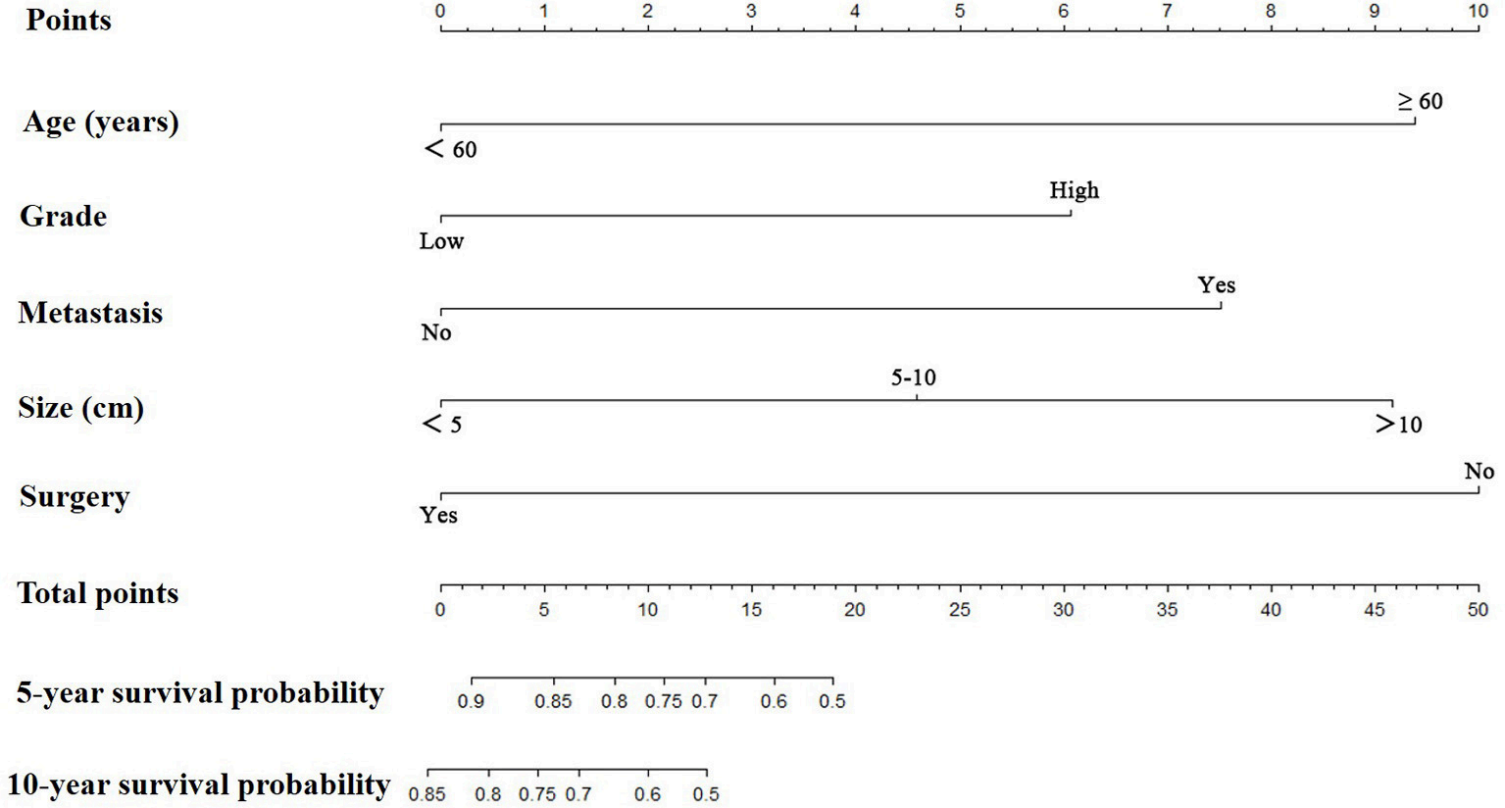
The graph shows the nomogram predicting 5- and 10-year overall survival of patients with extremity leiomyosarcoma. The nomogram summed the points identified on the scale for each predictor. The total points projected on the bottom scales indicate the probabilities of 5- and 10-year overall survival.

**Figure 2:**
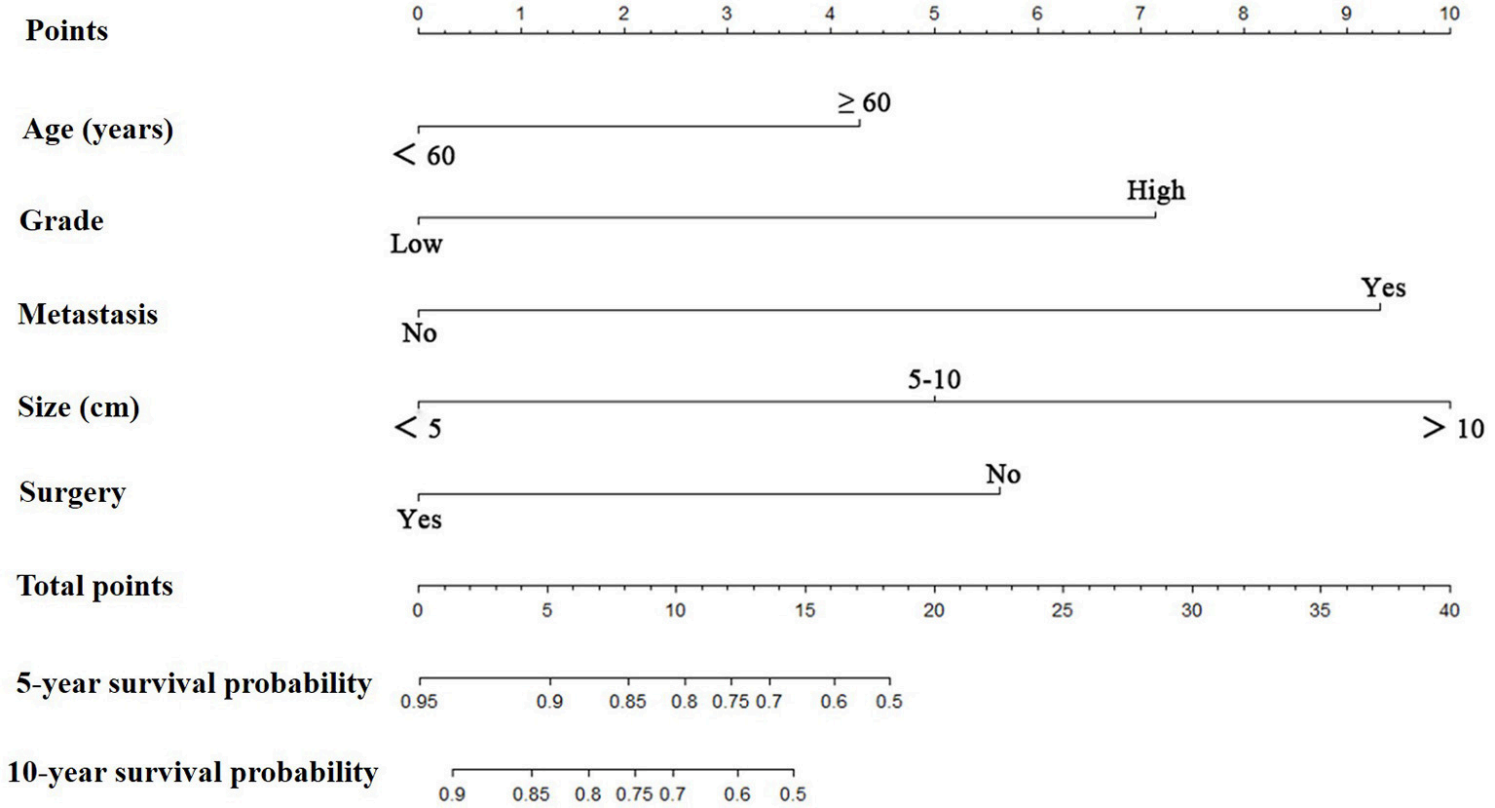
The graph shows the nomogram predicting 5- and 10-year cancer-specific survival of patients with extremity leiomyosarcoma. The nomogram summed the points identified on the scale for each predictor. The total points projected on the bottom scales indicate the probabilities of 5- and 10-year cancer-specific survival.

Prognostic nomograms for extremity LMS were constructed based on the five independent prognostic variables for OS and CSS from the training cohort (Figures 1, 2). The nomograms showed that surgery contributed most to OS and tumor size contributed most to CSS. To easily use the nomograms, one can add up the specific points of each predictor and draw a vertical line from the total score to the OS or CSS to estimate the prognosis of each patient. Detailed scores of each subtype of the variables are listed in Table 4. For example, a 50-year-old woman was diagnosed with high-grade extremity soft tissue LMS with a primary tumor size of 7.0 cm. She then underwent surgical treatment without metastasis. Adding up the points gave total scores of 10.7 and 12.1 points for the OS and CSS nomograms, respectively. Her estimated 5-year OS and CSS rates were 75 and 76%, respectively, according to the nomograms.

**Table 4:** Point assignment and prognostic score.

**Variable**	**OS nomogram**	**CSS nomogram**
**Age (Years)**
<60	0.0	0.0
≥60	9.4	4.3
**Tumor Grade**^**a**^
Low	0.0	0.0
High	6.1	7.1
**Distant Metastasis**
No	0.0	0.0
Yes	7.5	9.3
**Tumor Size**
<5 cm	0.0	0.0
5–10 cm	4.6	5.0
>10 cm	9.2	10.0
**Surgical Treatment**
Yes	0.0	0.0
No	10.0	5.6

a *Low: Grade I (well differentiated) and Grade II (moderately differentiated); High: Grade III (poorly differentiated) and Grade IV (undifferentiated anaplastic). OS, overall survival; CSS, cancer-specific survival*.

**Figure 3:**
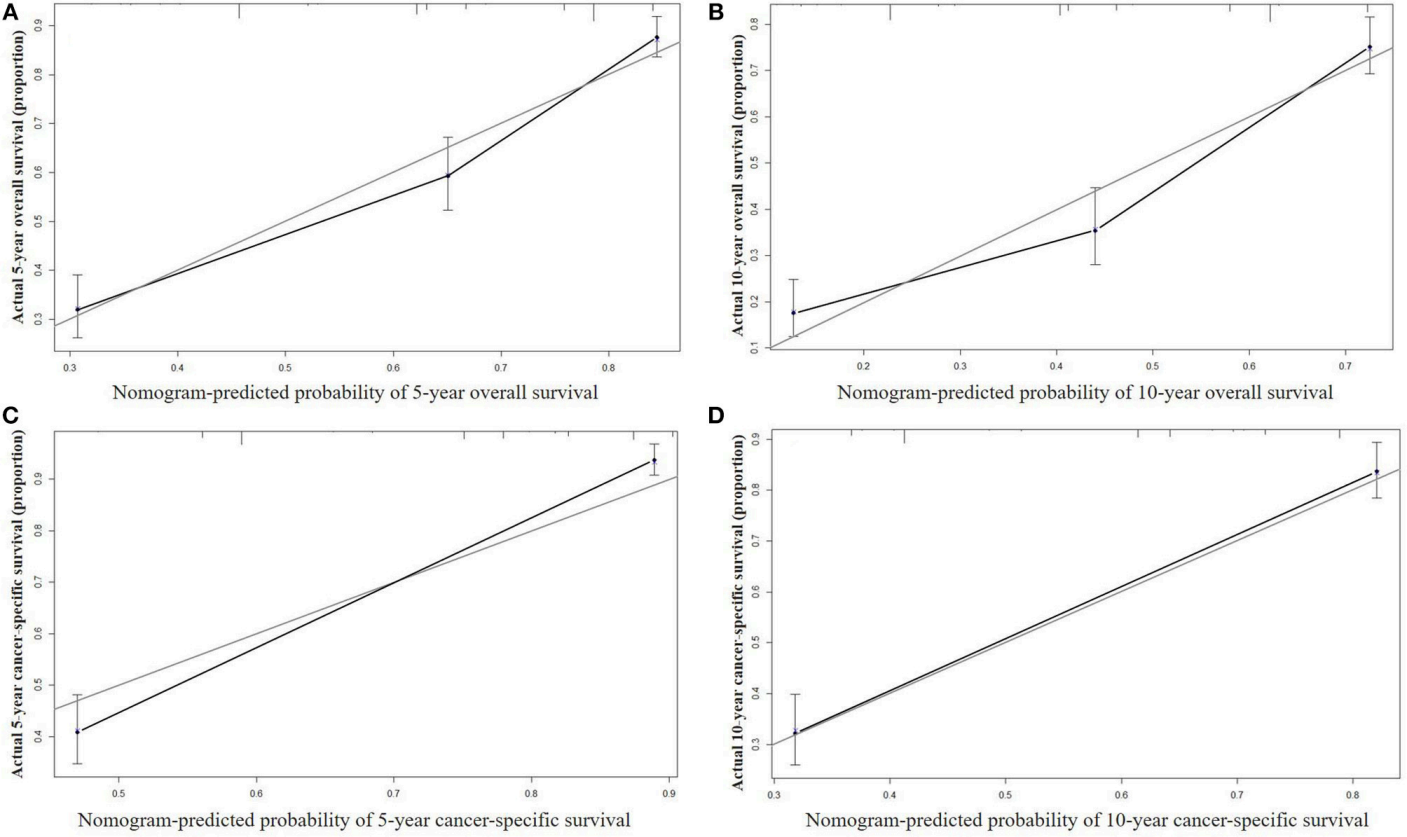
Internal calibration curves for 5-year **(A)** and 10-year **(B)** overall survival; and 5-year **(C)** and 10-year **(D)** cancer-specific survival. The X axis represents the nomogram predicted survival rate, whereas the Y axis represents the actual survival rate.

**Figure 4:**
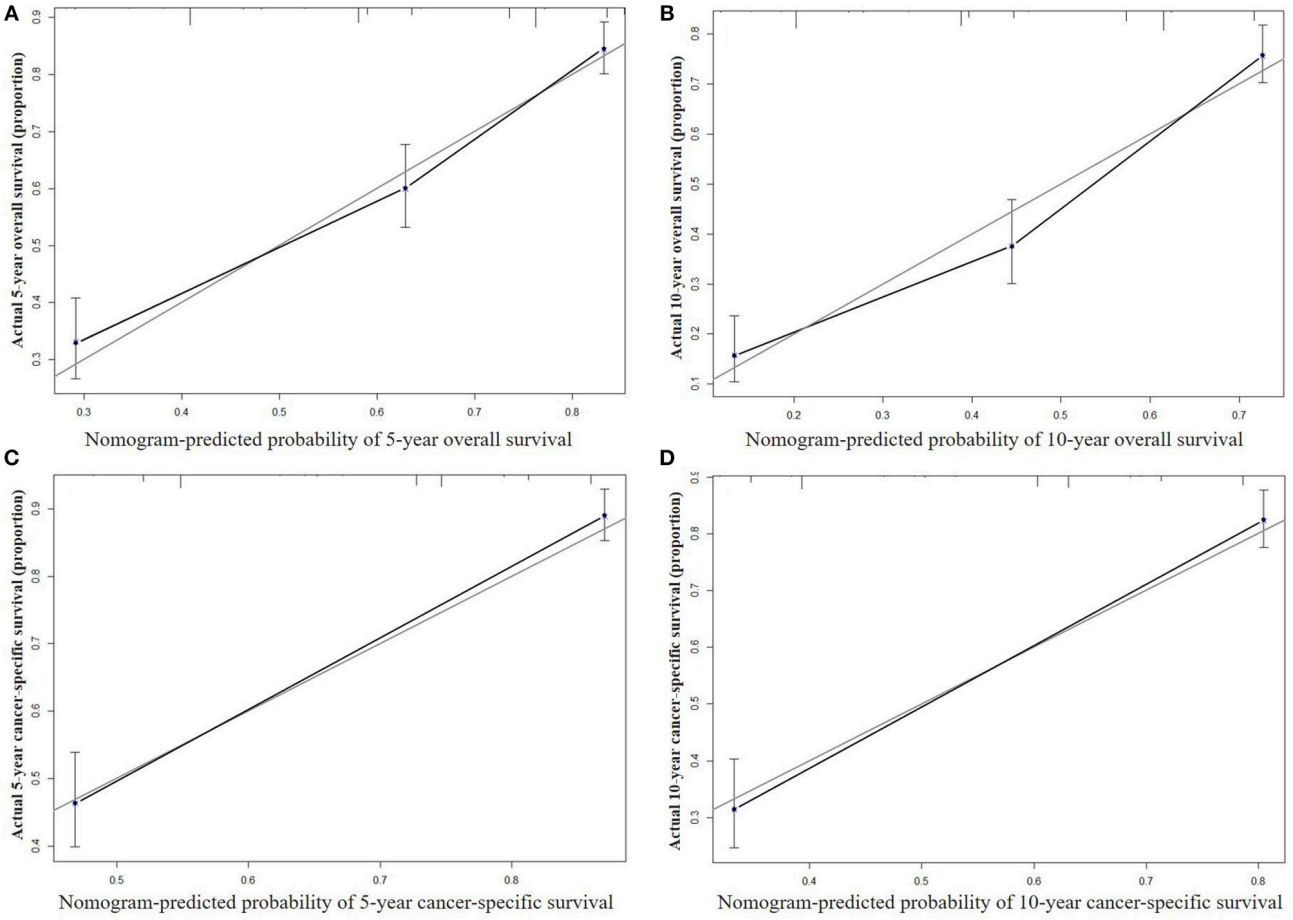
External calibration curves for 5-year **(A)** and 10-year **(B)** overall survival; and 5-year **(C)** and 10-year **(D)** cancer-specific survival. The X axis represents the nomogram predicted survival rate, whereas the Y axis represents the actual survival rate.

The nomograms were validated internally and externally. In the training cohort for internal validation, the C-index values for OS and CSS prediction were 0.776 (95% CI 0.752–0.801) and 0.835 (95% CI 0.810–0.860), respectively. In the validation cohort for external validation, the C-index values for OS and CSS prediction were 0.748 (95% CI 0.721–0.775) and 0.814 (95% CI 0.785–0.843), respectively. Excellent correlations between nomogram prediction and actual observation were indicated by the internal and external calibration curves for 5- and 10-year OS and CSS (Figures 3, 4).

## Discussion

Eligible patients (*n* = 1,528) with extremity soft tissue LMS were identified from the SEER database. Based on clinical characteristics and treatment information, we first established and validated prognostic nomograms for extremity soft tissue LMS. The proposed nomograms revealed excellent discrimination both internally and externally. Additionally, accurate survival predictions of the proposed nomograms were indicated by the calibration curves.

The nomogram is a pictorial display of prognosis prediction, which constitutes a significant part of clinical decision-making (22, 24). Nomograms provide rapid and simple prognostic information by integrating easily available and measurable variables. Thus, they represent a pragmatic tool for facilitating the popularization of patient consultation and individual treatment. Discrimination and calibration are used to confirm the presentation and validity of the nomogram.

In this study, nomograms were developed based on five easily accessible predictors, comprising age, tumor grade, metastasis, tumor size, and surgery. Multivariate analysis showed that age had a statistically significant association with OS and CSS, consistent with previous studies (14, 25, 26). One possible reason for this may be that older patients are more likely to have metastases. High histological grade has been identified as an indicator of a poor prognosis in somatic LMS (1, 7), consistent with our results. Despite intensive treatment, patients with extremity soft tissue sarcoma and metastasis usually have poor prognosis (1, 7, 27). Tumor size has been recognized as an important predictor of LMS (1, 28, 29). We identified tumor size ≥5 cm as an independent predictor for decreased OS and CSS in the extremity LMS population. However, no clear association between primary tumor site and survival was observed. Surgical resection is the main local treatment for extremity LMS patients and can prolong their survival (4, 11). In our study, surgical resection proved to be a significant independent predictor and resulted in superior survival. Although radiotherapy may alleviate pain and achieve good local control (30), it was not independently associated with OS or CSS in the current study.

Based on the independent predictors of survival that we confirmed, we constructed nomograms to maximize prognostic ability (Figures 1, 2). To use nomograms, the total points of all predictors should be correlated with the likelihood of survival at particular time intervals. Nomograms will be useful for clinicians to recommend certain instructions. More importantly, the proposed nomograms showed sufficient discriminatory power and accurate calibration. Nomograms have been constructed for other sarcomas and proved to be practical in their management (31–33). Zheng et al. (32) established nomograms by integrating age, tumor site, histology, tumor size, tumor grade, tumor stage, and surgery to accurately predict OS and CSS of osteosarcoma patients. Zhou et al. (33) developed a reliable and powerful nomogram to predict prognosis in Ewing sarcoma of bone. The proposed nomograms had sufficient discriminatory power and good consistency between the prediction and actual observation. These novel prognostic nomograms based on independent predictors enable surgeons to estimate individual survival probability and optimize treatment options.

There were some limitations to this study. First, the nomograms were developed using retrospective information from the database, potentially generating selection bias (34). Second, the nomograms did not include other clinical variables, such as site of involvement, radiotherapy, and chemotherapy, which might affect prognosis (17). Third, the SEER database does not document local recurrence rates after surgery or radiation therapy, which might influence survival. Moreover, the SEER database is publicly available and contains no personal identification information. Finally, the proposed nomograms are more relevant to the geographic registries from which the data were obtained.

## Conclusion

We first developed and validated prognostic nomograms for soft tissue extremity LMS, based on the SEER database. The established nomograms revealed excellent performance both internally and externally. This study provides a pragmatic tool that may help clinicians to better estimate the prognosis of patients with extremity LMS and to provide appropriate treatment recommendations. To generalize the use of the nomogram, further external validation is still required.

## Ethics Statement

This study followed standard guidelines and was approved by the Ethics Committee of the Second Affiliated Hospital of Jiaxing University. The SEER database are publicly available and it contains no personal identification information.

## Author Contributions

MX and GC contributed to conceptualization, formal analysis, review and editing, and project administration. JD and MX contributed to the methodology and investigation. GC and JH contributed to the software. JD and JH contributed to the validation. MX contributed to the original draft preparation and funding acquisition. All authors approved the final version of this manuscript to be published.

### Conflict of Interest Statement

The authors declare that the research was conducted in the absence of any commercial or financial relationships that could be construed as a potential conflict of interest.
